# Binocular Microscope

**Published:** 1853-03

**Authors:** 


*Binocular Microscope.*—At a meeting of the Physico-Medical Society, on Saturday evening, 2d October, Prof. J. L. Riddell called the attention of the society to an instrument of his own invention and manufacture, which promises to be of incalculable advantage in microscopic researches, especially in the prosecution of microscopic anatomy and physiology.

He remarked that he last year contrived, and had lately constructed and used, a combination of glass prisms, to render both eyes serviceable in microscopic observation. The plan is essentially as follows: Behind the objective, and as near thereto as practicable, the light is equally divided, and bent at right angles, and made to travel in opposite directions, by means of two rectangular prisms, which are in contact by their edges, that are somewhat ground away. The reflected rays are received, at a proper distance for binocular vision, upon two other rectangular prisms, and again bent at right angles, being thus either completely inverted, for an inverted microscope, or restored to their original direction. These outer prisms may be cemented to the inner by means of Canada Balsam; or left free to admit of adjustment to suit different observers. Prisms of other form, with due arrangement, may be substituted.

This method proves, according to Prof. Riddell's testimony, equally applicable to every grade of good lenses, from Spencer's best sixteenth, to a common three-inch magnifier, with or without oculars or erecting eye-pieces, and with a great enhancement of penetrating and defining power. It gives the observer perfectly correct views, in length, breadth and *depth*, whatever power he may employ; objects are seen holding their true relative positions, and wearing their real shapes. In looking at solid bodies, however, depressions sometimes appear as elevations, and *vice versa*, forming a curious illusion; for instance, a metal spherule may appear like a glass ball silvered on the under side, and the margin of a wafer may seem to ascend from the wafer into the air.

With this instrument, the microscopic dissecting knife can be exactly guided. The watchmaker and artist can work under the binocular eye-glass with certainty and satisfaction. In looking at microscopic animal tissues, the single eye may perhaps behold a confused amorphous or nebulous mass, which the pair of eyes instantly shape into delicate superimposed membranes, with intervening spaces, the thickness of which can be correctly estimated. Blood corpuscles, usually seen as flat disks, loom out as oblate spheroids. Prof. R. asserted, in short, that the whole microscopic world could thus be exhibited in a new light, acquiring a tenfold greater interest, displaying, in every phase, a perfection of beauty and symmetry indescribable.—*N. Orleans Monthly Med. Register.*

